# Examining the Impact of Storage Conditions on the Stability of a Liquid Formulation of mRNA-Loaded Lipid Nanoparticles

**DOI:** 10.3390/pharmaceutics17091194

**Published:** 2025-09-14

**Authors:** Mina Sato, Eleni Samaridou, Moritz Beck-Broichsitter, Masatoshi Maeki, Shunsuke Kita, Manabu Tokeshi, Katsumi Maenaka, Hideyoshi Harashima, Yusuke Sato

**Affiliations:** 1 Laboratory of Innovative Nanomedicine, Faculty of Pharmaceutical Sciences, Hokkaido University, Kita-12 Nishi-6, Kita-ku, Sapporo 060-0812, Japan; mina.sato@pharm.hokudai.ac.jp (M.S.); harasima@pharm.hokudai.ac.jp (H.H.); 2 Institute for Vaccine Research and Development (HU-IVReD), Hokkaido University, Kita-12 Nishi-6, Kita-ku, Sapporo 060-0812, Japan; 3 Merck KGaA, Frankfurter Str. 250, 64293 Darmstadt, Germany; eleni.samaridou@merckgroup.com (E.S.); moritz.beck-broichsitter@merckgroup.com (M.B.-B.); 4 Division of Applied Chemistry, Faculty of Engineering, Hokkaido University, Kita-13 Nishi-8, Kita-ku, Sapporo 060-8628, Japan; m.maeki@eng.hokudai.ac.jp (M.M.); tokeshi@eng.hokudai.ac.jp (M.T.); 5 Laboratory of Biomolecular Science and Center for Research and Education on Drug Discovery, Faculty of Pharmaceutical Sciences, Hokkaido University, Kita-12 Nishi-6, Kita-ku, Sapporo 060-0812, Japan; maenaka@pharm.hokudai.ac.jp (S.K.); kita@pharm.hokudai.ac.jp (K.M.); 6 Laboratory for Molecular Design of Pharmaceutics, Faculty of Pharmaceutical Sciences, Hokkaido University, Kita-12 Nishi-6, Kita-ku, Sapporo 060-0812, Japan

**Keywords:** lipid nanoparticles, mRNA delivery, storage stability, pH, temperature

## Abstract

**Background/Objectives**: This study investigated the effect of storage conditions on mRNA-LNPs in situ via identification of the formulation traits necessary for improving storage stability. **Methods**: We synthesized an ionizable lipid, namely TOT-28, which has a hydrolysis-susceptible ester bond in its hydrophilic head group that allows it to act as an indicator of the hydrophilic environment within the mRNA-LNPs. LNPs were stored either at 4 or 25 °C for up to 8 weeks to investigate the effect of pH and temperature on ester hydrolysis, internal mRNA integrity, physicochemical properties of the LNPs, and mRNA gene expression. **Results**: The results indicate that, at 25 °C, a lower buffer pH increases ester hydrolysis, whereas an opposite trend slightly occurs in ester hydrolysis with storage at 4 °C. We also found that TOT-28-based LNPs were less hydrated and microviscosity was higher at 4 °C compared with storage temperature at 25 °C. Therefore, TOT-28-based LNPs seem less sensitive to external buffer solutions because of a higher-order structure when stored at lower temperatures. In addition, we found that LNPs with different ionizable lipid structures exhibit distinct responses to pH changes at specific storage temperatures. **Conclusions**: Our findings provide novel insights into the appropriate conditions for long-term storage of the mRNA-LNPs as a liquid formulation.

## 1. Introduction

Lipid nanoparticles (LNPs) are sone of the most promising delivery platforms for RNA therapeutics and vaccines. LNPs have been used in Onpattro^®^, which was the first approved small-interfering RNA (siRNA) medicine, as well as for mRNA vaccines against SARS-CoV-2 (Comirnaty^®^, Spikevax^®^, and others) [1,2] and also against respiratory syncytial virus (mRESVIA^®^) [3]. LNPs have recently been employed to deliver RNA constructs (e.g., siRNA, microRNA, antisense oligonucleotide, self-replicating RNA, etc.) and are, nowadays, found in various stages of clinical development [4]. LNPs have shown great potential for use in RNA therapeutic immunotherapies for cancer, allergy tolerization, protein replacement therapies, and genome editing.

LNPs typically consist of four types of lipidic components ionizable lipids, cholesterol, phospholipids, and polyethylene glycol (PEG)-conjugated lipids. Ionizable lipids have an amino group with a positive charge that interacts with negatively charged RNA therapeutics under an acidic pH. Moreover, the positively charged ionizable lipids tend to destabilize endosomal membranes and promote endosomal escape followed by cytosolic release of RNAs. Therefore, numerous ionizable lipids have been developed in past years mainly to improve gene expression and to expand access to specific tissue and cells [5,6,7,8,9,10,11,12,13,14,15].

To gain a wider variety of applications, storage stability is a critical factor for LNP-based RNA therapeutics. The mRNAs are prone to inherent hydrolysis and oxidation [16,17]. Lipidic components are also affected by degradation and oxidation, which often results in the dysfunction of LNPs [18]. To this reason, both Comirnaty^®^ and Spikevax^®^ mRNA-vaccines required ultra cold storage temperatures (e.g., storage at −90 to −60 °C) and complex logistics, which inhibited their global distribution and access [19,20].

Looking into the underlying mechanisms, Parker et al. reported that ionizable lipids with tertiary amines generate fatty aldehyde impurities via *N*-oxidation followed by hydration [18]. These impurities covalently bind to mRNAs (i.e., adduct formation) inside LNPs and lead to a loss of activity during storage. Recently, Hashiba et al. demonstrated that piperidine-based ionizable lipids avoid adduct formation and greatly improve the storage stability of mRNA-LNPs at 4 °C as a liquid formulation [21].

The buffer composition can also affect the shelf-life of mRNA-LNPs. Henderson et al. investigated commonly used biological buffers such as HEPES, Tris, and phosphate-buffered saline (PBS) to determine the impact they exert on the structural changes and transfection efficiency of mRNA-loaded DLin-MC3-DMA (MC3)-LNPs and found that Tris and HEPES yield better cryoprotection and transfection efficiency compared with that of PBS [22]. It also is important to mention that after the buffer formulation in Comirnaty^®^ was changed from PBS to Tris buffer [23], regulatory agencies increased the limit of Comirnaty’s prolonged storage time from 4 to 10 weeks under refrigerated conditions. Tris buffer is reported to capture lipid-derived aldehyde impurities and, thus, to reduce mRNA-lipid adduct formation [24].

Water inside LNPs is a key player in the hydrolysis of both lipidic components and mRNAs. Alteta et al. proposed two shell models based on their analysis of mRNA-loaded MC3-LNPs by means of small-angle X-ray scattering (SAXS), cryogenic transmission electron microscopy (cryoTEM), and small-angle neutron scattering (SANS). This allowed them to estimate that the water volume fraction in the core of the LNPs should be approximately 25% [25]. Lyophilization is one of the promising strategies to reduce the residual water inside LNPs and protect mRNAs and lipids from hydrolysis [25,26,27,28,29]. However, several studies have reported that different LNP formulations seem to favor different optimal conditions of temperature, cryoprotectant, and buffer [30,31,32]. This attractive method, however, is an expensive and complicated process that potentially induces structural changes in mRNA-LNPs via reconstruction. Therefore, long-term storage of the mRNA-LNPs as a liquid formulation is desired for use worldwide. It is crucial to understand how storage conditions affect the hydrophilic environment inside the mRNA-LNPs when suspended in a buffer solution.

In this study, we synthesized an ionizable lipid, namely TOT-28, with a hydrolysis-susceptible ester bond in its hydrophilic head group. This allowed us to in situ monitor the hydrophilicity of the microenvironment around its head group inside the mRNA-LNPs. To establish the integrity of mRNA-LNPs upon storage, the microviscosity and hydration degree of the mRNA-loaded LNPs were measured as a function of storage pH and temperature to unveil the relationships between physical properties of the LNP membranes and storage stability of the mRNA-LNPs. The results indicated that the buffer pH affects the hydrophilic environment inside mRNA-LNPs in different manners depending on the storage temperature, which suggests interaction between temperature and pH. Our findings provide novel insights into the appropriate conditions for long-term storage of the mRNA-LNPs as a liquid formulation.

## 2. Materials and Methods

### 2.1. Materials

A pH-sensitive cationic lipid, TOT-28, was synthesized as described in the Supplementary Information. MedChemExpress (Monmouth Junction, NJ 08852, USA) supplied the DLin-MC3-DMA (MC3) (catalog No. HY-112251) and ALC-0315 (ALC) (catalog No. HY-138170). Cholesterol (chol) was purchased from SIGMA Aldrich (St. Louis, MO, USA, catalog No. C8667-25G). The NOF Corporation (Tokyo, Japan) supplied 1,2-distearoyl-*sn*-glycero-3-phosphocholine (DSPC, catalog No. MC-8080) and PEG_2k_-DMG (catalog No. GM-020). Ribogreen was purchased from Molecular Probes (Eugene, OR, USA, catalog No. R11491). MedChemExpress (Monmouth Junction, NJ, USA) supplied the 2-(*p*-toluidino)-6-napthalene sulfonic acid (TNS) (catalog No. HY-W250727). DCVJ was purchased from SIGMA Aldrich (St. Louis, MO, USA, catalog No. 72335). The 6-dodecanoyl-2-dimethylaminonaphthalene (Laurdan) was purchased from invitrogen (Eugene, Oregon, USA, catalog No. D250). Firefly luciferase (Fluc)-encoding mRNA (CleanCap, 5moU modified) was purchased from Trilink BioTechnologies (San Diego, CA, USA, catalog No. L-7202). An iLiNP microfluidic device was fabricated and used for microfluidic mixing, as previously described [33,34].

### 2.2. Preparation of mRNA-Loaded LNPs

The mRNA-loaded LNPs were prepared, as previously described [34]. Briefly, an ethanol solution containing an ionizable lipid, DSPC, chol, and PEG_2k_-DMG at Mol% ratios of 50/3/47/1.5, respectively, was prepared to reach a total lipid concentration of 8 mM. The FLuc-mRNA was dissolved in 50 mM of citrate buffer (pH 4.0). The lipid mixture was combined with a mRNA aqueous solution using a glass-based iLiNP device at a total flow rate of 2 or 5 mL/min with a flow-rate ratio (RNA to lipid) of 3. The nitrogen-to-phosphate ratio was adjusted to 6. Syringe pumps (Harvard apparatus, MA, USA) were used to control the flow rate of the solutions. The resultant LNP solution was then dialyzed against 20 mM of Tris-HCl buffer (pH7.0) containing 9% (*w*/*v*) sucrose using a Slide-A-Lyzer MINI Dialysis Device (molecular weight cut-off MWCO 10 kDa) (Thermo Fisher Scientific, Rockford, IL, USA, catalog No. A52977) for 2 to 24 h in order to reconstruct the LNP structure by neutralization. The LNPs were concentrated via ultrafiltration using an Amicon Ultra-15 unit (MWCO 100 kDa) (Merck Millipore Ltd., Carrigtwohill, Co. Cork, Ireland, UFC910096) as needed. The LNP solution was then dialyzed against citrate-Tris buffer (pH 5.0, 5.5, 6.0, 6.5, or 7.0) containing 9% (*w*/*v*) sucrose. Sucrose was used to make the storage buffer isotonic.

### 2.3. Characterization of the LNPs

The ζ-average size, polydispersity index (PdI), and ζ-potential of the LNPs were measured using a Zetasizer Nano ZS ZEN3600 instrument (Malvern Instruments, Worcestershire, UK). To reduce the influence of storage buffer pH during ζ-potential measurement, all LNPs were diluted 250-fold with 10 mM HEPES buffer (pH 7.4). The encapsulation efficiency and total concentration of mRNA were measured by Ribogreen assay, as described previously [34].

The apparent pKa of the TOT-28-LNPs was determined by TNS assay. Briefly, 30 μM of LNP lipids and 6 μM of TNS were mixed in 50 μL of 20 mM citrate buffer, 20 mM sodium phosphate buffer, or 20 mM Tris-HCl buffer, containing 130 mM NaCl, at pH values ranging from 3.5 to 9.5 in a black 384-well plate by means of an epMotion 5070 (Eppendorf SE, Hamburg, Germany). Fluorescence (excitation and emission wavelength of 321 and 445 nm, respectively) was read using a microplate reader (Varioskan Lux, Thermo Fisher Scientific, Waltham, MA, USA) at 37 °C. The pKa value was determined as the pH at which the half-maximum fluorescence intensity was reached.

### 2.4. Animals

BALB/c mice (female, 5–6 weeks old) were sourced from Japan SLC (Shizuoka, Japan). Mice were maintained on a regular 12 h light/12 h dark cycle in a specific animal facility at Hokkaido University. Four to five mice were housed in each cage. The mice were fed a pelleted mouse diet (cat# 5053, LabDiet, Gray Summit, MO, USA) and water was available ad libitum.

### 2.5. Measurement of Fluc Activity Using IVIS

Mice were injected with Fluc mRNA-loaded LNPs at a dose of 5 µg mRNA/mouse into the caudal thigh muscle (50 μL injection volume). At 20 h post-injection, bioluminescence imaging was performed using an in vivo imaging system (IVIS Lumina III, PerkinElmer, Waltham, MA, USA) 20 min after intraperitoneal injection of D-luciferin at a dose of 150 mg/kg under anesthesia with three types of mixed anesthetic agents: 0.3 mg/kg of medetomidine, 4.0 mg/kg of midazolam, and 5.0 mg/kg of butorphanol.

### 2.6. Micro Viscosity of the Lipid Nanoparticles Membranes

DCVJ labeling of the LNPs was performed by adding 0.5 mM of DCVJ dissolved in DMSO to the mRNA-loaded LNP solution (1 mM as a total lipid concentration) to yield a lipid: DCVJ molar ratio of approximately 400:1. For the fluorescence measurements, 200 μL of the DCVJ-labeled LNPs (1 mM and 2.5 μM as total lipid and DCVJ, respectively) were added to black 96-well plates. The plates were incubated for 30 min or more at either 4 or 25 °C and fluorescence spectra (excitation and emission wavelength of 440 nm and 460–650 nm with 2 nm interval, respectively) were then measured using a microplate reader (Varioskan Lux, Thermo Fisher Scientific, Waltham, MA, USA). To standardize the DCVJ concentration, the DCVJ-derived absorption spectra (350–550 nm with 2 nm interval) were also measured after solubilizing the LNPs by adding 20 μL of 10% sodium dodecyl sulfate (FUJIFILM Wako Pure Chemical Corporation, Osaka, Japan) followed by incubation at 60 °C for 10 min with gentle mixing at 700 rpm. To estimate the viscosity, R (fluorescence to absorption ratio) was calculated following a method described in the literature [35]. Specifically, the integral of the fluorescence spectrum from 460 to 650 nm was divided by the integral of the absorption spectrum from 400 to 510 nm.

### 2.7. Hydration Degree of the Lipid Nanoparticle Membrane

Laurdan labeling of the LNPs was performed by adding 1 mM of laurdan dissolved in DMSO to the lipid mixture (8 mM as a total lipid concentration) in ethanol to yield a lipid/laurdan molar ratio of 500:1 before formulation. For the fluorescence measurements, 200 μL of the laurdan-labeled LNPs (0.1 mM as a total lipid concentration, 0.2 μM as laurdan) was added to black 96-well plates. The plates were incubated for 30 min or more at either 4 or 25 °C and fluorescence was then measured using a microplate reader (Varioskan Lux, Thermo Fisher Scientific, Waltham, MA, USA). The samples were excited at 340 nm and the fluorescence spectra from 360 to 600 nm was obtained. To estimate the hydration degree of the LNP membrane, the generalized polarization (GP) value was calculated using the following formula:

GP = (*I_440_* − *I_490_*)/(*I_440_* + *I_490_*), (1)

In that formula, *I_440_* and *I_490_* are the fluorescence intensities at emission wavelengths of 440 and 490 nm, respectively.

### 2.8. Capillary Gel Electrophoresis

Capillary electrophoresis was performed using an QIAxcel Connect capillary gel electrophoresis system (QIAGEN, Hilden, Germany). The sample preparation was as follows. First, 100 µg mRNA/mL of mRNA-LNP samples was diluted to 5 ng/µL in QX RNA HS Dilution buffer^®^. The mRNA-LNP sample was then added to an equal volume of 5% (*w*/*v*) tritonX-100 (SIGMA-ALDRICH, St. Luis, MO, USA) in QX RNA HS Dilution buffer^®^. The mixture was incubated at room temperature for 10 min or more. One µL of the mixture was mixed with 1 µL of QX RNA Denaturation buffer^®^, heated at 70 °C for 2 min, and then chilled at 4 °C for at least 5 min using a thermal cycler (MiniAmp Thermal Cycler, Thermo Fisher Scientific,Waltham, MA, USA). The samples were diluted with 8 µL of QX RNA Booster buffer^®^ and then transferred to a PCR tube for capillary electrophoresis analysis. The electropherogram for each injection was transferred to QIAxcel Screengel 2.0 software and Excel for analysis. The data calculation was based on the area under the curve of the sample line and above the base line. To define the peak derived from fragmented mRNA, we defined the mRNA fragment area as the region from A to B. The total mRNA area was defined as the region from A to reach the final retention time of measurement. The percentage of fragmented mRNA was calculated as the ratio of fragmented mRNA/total mRNA.

A: The retention time elapsed at 3 times the half-width of the half maxima of the internal standard marker peak.

B: The retention time was faster than 3 times the half-width at the half maxima of the intact mRNA peak.

### 2.9. Measurement of TOT-28 Hydrolysis Using LC/ELSD

To measure the TOT-28 in TOT-28-LNPs, the LNP samples were diluted to 4 mM of total lipid using a 9% (*w*/*v*) sucrose solution. Sixty µL of the diluted samples were extracted with 240 µL of acetonitrile/isopropanol (*v*/*v* 1:2). Following centrifugation (4 °C, 15,000× *g*, 10 min), the supernatant was filtered, and the filtrate was analyzed using an LCMS-2050 (Shimazu Corporation, Kyoto, Japan) with an ELSD-LT III detector (Shimazu Corporation). Separation was carried out using a Shim-pack Arata C18 Column (pore size: 120 Å; particle size: 5 µm; inner diameter: 50 mm; length: 2.0 mm) at a column temperature of 60 °C. Mobile phase A comprised 5 mM of ammonium acetate and mobile phase B consisted of isopropanol/acetonitrile (2:1) and 5 mM of ammonium acetate. Separation was accomplished using a step gradient with an initial 0 min hold at 60% of B, a 2 min gradient from 60 to 75% of B, a 4 min gradient from 75 to 98% of B, and a hold at 98% of B, which was delivered at a flow rate of 0.2 mL/min with an injection volume of 5 µL.

### 2.10. Cryogenic Transmission Electron Microscopy (Cryo-TEM)

Freshly prepared mRNA-loaded TOT-28-LNPs exposed to the described pH and temperature conditions were added to Quantifoil R1.2/1.3 Cu 300 grids at 4 or 25 °C, and frozen in liquid ethane using FEI Vitrobot MarkIV. The grids were stored in liquid nitrogen until used for imaging. Glacios Cryo TEM (Thermo Fisher Scientific Inc., Waltham, MA, USA) was used to obtain the images. The instrument was operated at 200 kV. Samples were imaged at a 73,000× magnification.

### 2.11. Data Analysis

Results are expressed as the mean ± the standard deviation (SD) of independent experiments. For in vitro experiments, technically independent experiments were conducted using LNP prepared each time. For comparisons between multiple groups, two-way analysis of variance (ANOVA) was performed followed by a Tukey post hoc test. Group size, definition of center, and dispersion and precision measures are also noted in the figure legends as appropriate. All graphs were prepared using GraphPad Prism software version 10.1.2 (San Diego, CA, USA).

## 3. Results

### 3.1. Characterization of TOT-28-LNPs

**Figure 1:**
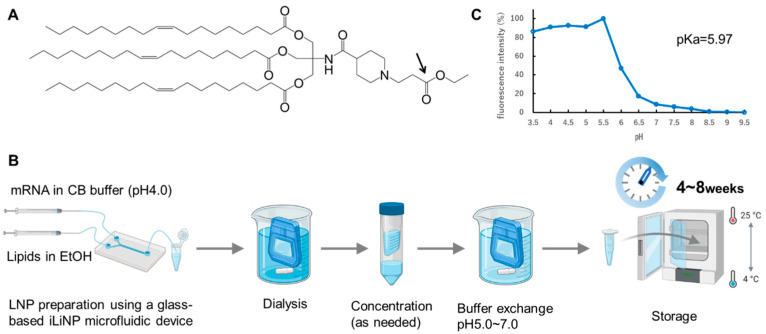
Examination of the storage stability of TOT-28-LNPs as a liquid formulation. (**A**) Chemical structure of TOT-28. The arrow indicates an expected hydrolyzing bond. (**B**) Flow chart for preparation and storage of TOT-28-LNPs. CB: citrate buffer. (**C**) Apparent p*K*_a_ value of the TOT-28-LNPs measured via TNS assay.

TOT-28 consists of three oleic acid-derived hydrophobic scaffolds and a piperidine *N*-ethylpropionate structure as the hydrophilic head group (Figure 1A). The piperidine structure was adopted to minimize adduct formation followed by mRNA inactivation [21]. The *N*-ethylpropionate moiety was introduced to monitor the microenvironment around the hydrophilic region of ionizable lipids in mRNA-LNPs under different storage conditions. The *N*-ethylpropionate moiety is susceptible to hydrolysis under both acidic and basic conditions. Its hydrolysis susceptibility is affected by the degree of hydration around the ester bond. A parental TOT-28 and its hydrolyzed product could be quantified following separation by reverse-phase high-performance liquid chromatography (HPLC) based on the difference in hydrophobicity. Therefore, TOT-28 has a suitable structure for revealing the differences in the microenvironment around the hydrophilic group of ionizable lipids under various storage conditions.

**Figure 2:**
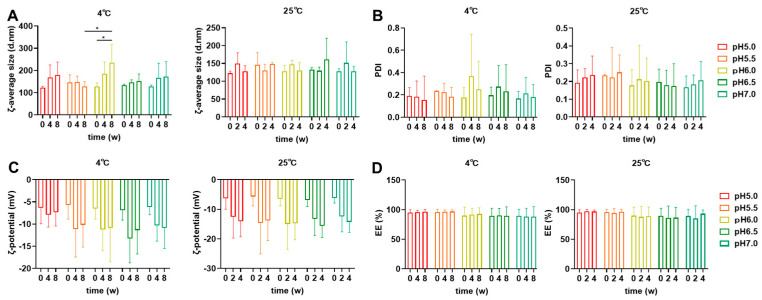
Physicochemical properties of the TOT-28-LNPs during storage at different values for pH and temperature. ζ-Average size (**A**), polydispersity index (PDI) (**B**), ζ-potential (**C**), and percentage of mRNA encapsulation efficiency (EE%) (**D**) of the TOT-28-LNPs were measured after storage as a liquid formulation with the indicated storage conditions and timepoints. n =  3 technically independent measurements per each condition. Two-way ANOVA was performed followed by a Tukey test. * *p* < 0.05. Statistically insignificant results are not included.

TOT-28-LNPs were assembled by vigorously mixing TOT-28, cholesterol (Chol), 1,2-distearoyl-*sn*-glycero-3-phophocholine (DSPC), and 1,2-dimyritoyl-*rac*-glycero-3-methoxypolyethylene glycol-2000 (PEG_2k_-DMG) at a fixed molar ratio with Firefly luciferase (FLuc) mRNA using a microfluidic device followed by purification with dialysis (Figure 1B). The TOT-28-LNPs showed an apparent acid dissociation constant (p*K*_a_) value of 5.97 (Figure 1C). The physicochemical properties of the TOT-28-LNPs during storage at different values of pH (ranging from 5.0 to 7.0) and temperature (4 or 25 °C) were monitored (Figure 2). The diameters of the fresh TOT-28-LNPs were 123 to 133 nm (Figure 2A). The polydispersity index (PDI) of these fresh TOT-28-LNPs ranged from 0.17 to 0.23 (Figure 2B). The diameter and PDI gradually increased over the storage period independently of storage temperature and pH. The ζ-potentials of these fresh TOT-28-LNPs were −5.7 to −6.9 mV (Figure 2C). At 4 °C, the ζ-potential slightly decreased to −7.3 mV at the final timepoint at pH 5.0. On the other hand, the ζ-potential dropped significantly to a range of −10.2 to −11.4 mV at other values of pH. Furthermore, the ζ-potential decreased dramatically to within a range of −13.8 to −15.5 mV regardless of the pH values at 25 °C. Regardless of pH value, the encapsulation efficiency (EE) was constant over the storage period without depending on the storage conditions (Figure 2D). The EE of TOT-28-LNPs at pH 5.0 and 5.5 were approximately 95%, which was approximately 5% higher than that at higher pH.

### 3.2. Ester Hydrolysis of TOT-28

**Figure 3:**
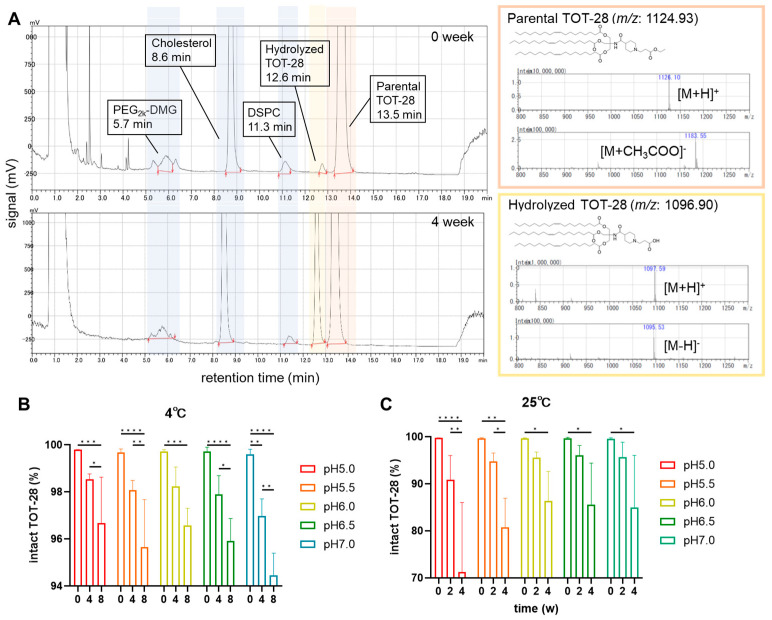
Ester hydrolysis of TOT-28 measured by means of LC/ELSD. (**A**) Typical chromatograph and retention time of each LNP component extracted from the TOT-28-LNPs stored at pH 7.0 at 25 °C. The chemical structures of parental (orange box) and hydrolyzed TOT-28 (yellow box) and the MS spectra in positive mode (upper) and negative mode (lower) are shown on the right. (**B**,**C**) Percentage of intact TOT-28 stored at 4 °C (**B**) and 25 °C (**C**). n =  3 technically independent measurements per each condition. Two-way ANOVA was performed followed by a Tukey test. * *p* < 0.05, ** *p* < 0.01, *** *p* < 0.001, **** *p* < 0.0001. Statistically insignificant results are not included.

The degree of the hydrolysis of an ester bond in the hydrophilic region of TOT-28 was monitored using a HPLC setup equipped with an evaporative light scattering detector (LC/ELSD) over the storage period (Figure 3). Retention time for the peak derived from hydrolyzed TOT-28 was 12.6 min, which was clearly separated from that of the parental TOT-28 observed at 13.5 min (Figure 3A). These peaks were assigned based on mass spectrometry (MS) analysis following consistent LC conditions. Under these LC/ELSD conditions, other potential hydrolyzed TOT-28 products lacking a hydrophobic scaffold were not detected throughout the experiments, which indicates that the ester bond in the hydrophilic region is highly susceptible to hydrolysis compared with that in the scaffold, as expected. The peak derived from the parental TOT-28 product was expressed as the relative percentage of the total peaks derived from all TOT-28 samples (i.e., sum of the parental and the hydrolyzed product) (Figure 3B,C). At 25 °C, the hydrolytic rate of the TOT-28 increased as the pH dropped to within a range of pH 6.0 or less and the parental TOT-28 was reduced to 70% after 4 weeks of storage at pH 5.0 (Figure 3C). By contrast, more than 94% of the TOT-28 was maintained intact at 4 °C after 8 weeks of storage across the range of tested pH. Interestingly, less of the TOT-28 was hydrolyzed at lower pH at 4 °C, which could indicate a potentially opposite tendency from that observed at 25 °C.

### 3.3. Degradation of mRNA Inside TOT-28-LNPs

**Figure 4:**
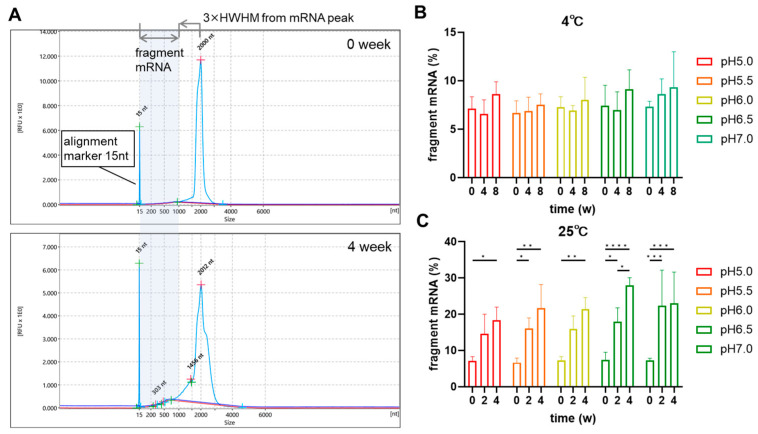
Analysis of the integrity of mRNAs in the TOT-28-LNPs by means of capillary gel electrophoresis. (**A**) Typical electropherogram of mRNA extracted from the TOT-28 LNPs stored at pH 7.0 at 25 °C. The defined regions for fragmented mRNA are highlighted by the light blue background. (**B**,**C**) Percentage of fragmented mRNA stored at 4 °C (**B**) and 25 °C (**C**). n =  3 technically independent measurements per each condition. Two-way ANOVA was performed followed by a Tukey test. * *p* < 0.05, ** *p* < 0.01, *** *p* < 0.001, **** *p* < 0.0001. Statistically insignificant results are not included.

The degree of mRNA degradation in the TOT-28-LNPs was monitored during storage by means of capillary electrophoresis (Figure 4). The % of fragmented mRNA in the TOT-28-LNPs was expressed as a relative percentage of the total peaks (i.e., sum of both intact and fragmented mRNA), which was approximately 7% in all fresh LNPs (Figure 4B,C). Whereas only a slight increase to below 10% was observed at 4 °C over 8 weeks (Figure 4B), a clear increase of up to 30% was observed at 25 °C over 4 weeks (Figure 4C). The degree of increase at higher pH tended to become larger at 25 °C.

### 3.4. Hydration Degree and Microviscosity of LNP Lipid Membranes

**Figure 5:**
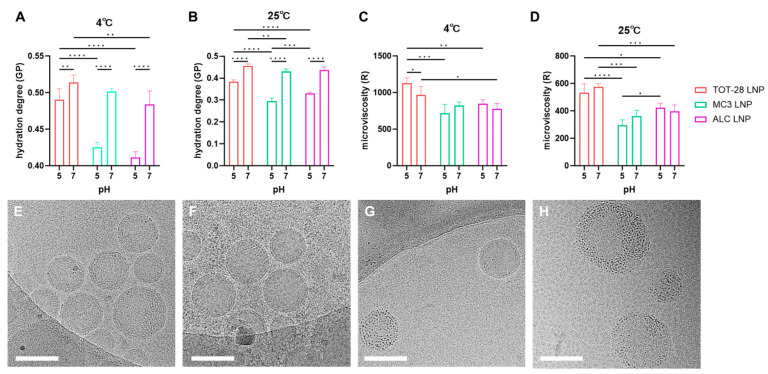
Physicochemical properties and structure of the mRNA-loaded LNPs under different storage conditions. (**A**,**B**) Hydration degree of the LNPs measured using laurdan at 4 °C (**A**) and 25 °C (**B**). n =  3 technically independent measurements per each condition. Two-way ANOVA was performed followed by a Tukey test. ** *p* < 0.01, *** *p* < 0.001, **** *p* < 0.0001. Statistically insignificant results are not included. (**C**,**D**) Microviscosity of the LNPs measured using DCVJ at 4 °C (**C**) and 25 °C (**D**). n =  3 technically independent measurements per each condition. Two-way ANOVA was performed followed by a Tukey test. * *p* < 0.05, ** *p* < 0.01, *** *p* < 0.001, **** *p* < 0.0001. Statistically insignificant results are not included. (**E**–**H**) CryoTEM observation of TOT-28-LNPs exposed to different pH and temperature conditions. (**E**) pH 7 and 4 °C, (**F**) pH 7 and 25 °C, (**G**) pH 5 and 4 °C, (**H**) pH 5 and 25 °C. Scale bars represent 100 nm.

Based on the above observation that storage temperature changed the effect of pH on the ester hydrolysis of TOT-28, both hydration degree and microviscosity of the fresh TOT-28-LNPs at different pH (5.0 and 7.0) and temperature (4 and 25 °C) were measured to understand the impact of the storage conditions on the physicochemical microenvironment of the lipid membranes. The LNPs consisting of commercially available ionizable lipids DLin-MC3-DMA (MC3) and ALC-0315 (ALC) were also used for comparison. The hydration degree of the LNPs was measured using laurdan and is expressed using common generalized polarization (GP) values. Under all conditions, TOT-28 had a higher GP value and was more hydrophobic than other ionizable lipids. Regardless of the differences in ionizable lipids, the GP values dropped at higher temperature and lower pH, indicating a more disordered lipid bilayer in acidic environments and with higher temperatures (Figure 5A,B). However, the decrease in the GP values of the TOT-28-LNPs caused by a drop in pH was clearly lower than that for others. The ratios of GP values at pH 5.0 and at pH 7.0 were 0.95 (TOT-28), 0.85 (MC3), and 0.85 (ALC) at 4 °C compared with 0.84 (TOT-28), 0.68 (MC3), and 0.76 (ALC) at 25 °C, respectively. These results indicate that the hydration of TOT-28-LNPs was less affected by pH change compared with that of others. In addition, the degree of hydration measured by laurdan and the degree of hydrolysis of TOT-28 did not match, which was thought to be due to the fact that the localization of the fluorescent indicator and specific LNP lipids (ionizable lipids in this case) did not match.

The microviscosity of LNPs was measured using 9-(2,2-dicyanovinyl) julolidine (DCVJ) and is expressed as the R value, which is the integrated fluorescence emission-to-absorbance ratio. Regardless of the differences in ionizable lipids, the R values at 4 °C were higher than those at 25 °C under the same pH (Figure 5C,D). The TOT-28-LNPs showed the highest R values under all storage conditions. Interestingly, the trends in microviscosity between pH values of 5.0 and 7.0 were clearly different between the three ionizable lipids. The MC3-LNPs showed a lower microviscosity at pH 5.0, whereas the ALC-LNPs showed a slightly higher microviscosity at pH 5.0 for both temperatures. Furthermore, the TOT-28-LNPs showed a decreasing trend with a decrease in the pH at 4 °C and the opposite trend at 25 °C. These results show that differences in the chemical structures of ionizable lipids not only alter the degree of microviscosity between lipids, but the responses to changes in storage temperature and pH are also affected. It is difficult to clearly explain why the change in microviscosity according to storage conditions differs depending on the ionizable lipid, but it may reflect differences in the internal structure of LNPs due to differences in the three-dimensional structure of ionizable lipids. In any case, the fact that ionizable lipids exhibit different changes in physicochemical properties of LNPs depending on storage conditions emphasizes the risk of a uniform understanding of LNP properties and the importance of individual verification. Next, we observed the particle structure of TOT-28-LNPs exposed to different pH and temperature conditions using cryo-TEM (Figure 5E–H). The TOT-28-LNPs exhibited a distinct lamellar structure in the outer layer and an amorphous structure in the core at pH 7 (Figure 5E,F). This is consistent with the core–shell model proposed by Arteta et al., which features a shell layer composed primarily of DSPC [25]. Interestingly, at pH 5, which is lower than the apparent pKa of TOT-28, numerous dot-like high-electron-density nanostructures with diameters of approximately 3–5 nm were observed within the LNPs (Figure 5G,H). This is highly similar to the “electron-dense gritty texture of LNP cores reflecting lipid-bound, condensed-state mRNA mingled with the excess of ionizable lipids” suggested by Szebeni [36], and “the high-electron-density mottles associated with the bodies of the LNPs” observed by Brader [37]. Furthermore, TOT-28-LNPs contain a greater number of dot-like high-electron-density nanostructures compared with the aforementioned previous studies that examined Comirnaty or LNPs containing Moderna’s proprietary ionizable lipids, which may be attributed to differences in the three-dimensional structure of the ionizable lipids. In addition, at pH 5, the lamellar structure of the LNP surface is either masked or has disappeared, which suggests the redistribution of protonated TOT-28 to the surface. These observations suggest that the pH of the external aqueous phase significantly affects the structure of internal mRNA, the arrangement of ionizable lipids, and the microenvironment surrounding each molecule. No obvious structural changes in LNPs were observed at each pH in response to storage temperature. However, the distinct LNP structures at different pH values could account for the opposite signs of microviscosity shift in response to pH changes at temperatures between 4 and 25 °C, and further detailed investigation is desirable.

### 3.5. In Vivo Expression of FLuc-mRNA in TOT-28-LNPs

**Figure 6:**
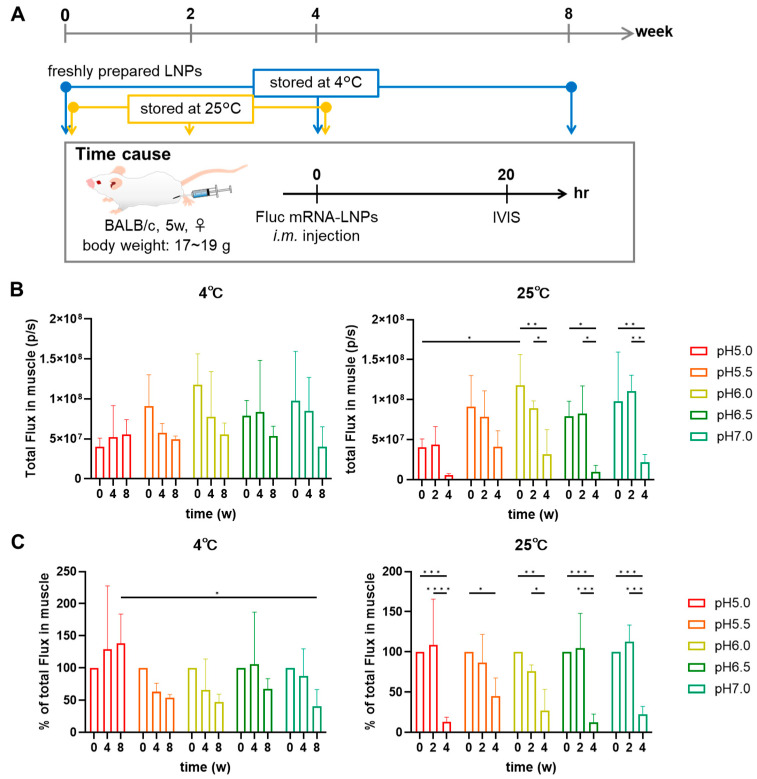
Gene expression activity of the mRNA-loaded LNPs following storage under different conditions and times. (**A**) Timeline of the in vivo study to evaluate storage stability of each LNP. In Vivo imaging system (IVIS) measurements were performed following the storage of LNPs for the indicated times, temperatures, and pH. n =  3 biologically independent mice per group. Two-way ANOVA was performed followed by a Tukey test. Statistically insignificant results are not included. (**B**,**C**) Absolute value (**B**) and relative value against week 0 (**C**) of bioluminescence (total flux) at the injection site (caudal thigh muscle), as measured via IVIS. n =  3 biologically independent mice per group. Two-way ANOVA was performed followed by a Tukey test. * *p* < 0.05, ** *p* < 0.01, *** *p* < 0.001, **** *p* < 0.0001. Statistically insignificant results are not included.

To reveal the impact of storage conditions on the in vivo performance of LNPs, bioluminescent levels at the injection site (caudal thigh muscle) were quantified 20 hrs following an intramuscular injection of TOT-28-LNPs following storage under indicated conditions and time periods. Total flux of the fresh TOT-28-LNPs at different levels of pH varied from 4 × 10^7^ to 12 × 10^7^ p/s with the lowest level recorded at pH 5.0 (Figure 6A). At 4 °C, the gene expression level of the LNPs stored at pH 5.0 was maintained for up to 8 weeks whereas that stored at pH 5.5 or above dropped to approximately 50% of the initial levels (Figure 6C). At 25 °C, on the other hand, gene expression levels were maintained for 2 weeks but clearly dropped to less than 50% of the initial levels at 4 weeks irrespective of the storage pH (Figure 6C), probably due to hydrolysis of both TOT-28 and mRNA.

## 4. Discussion

In this study we focused on analyzing the effects that both storage temperature and pH exert on the hydrophilic environment inside mRNA-LNPs in situ in terms of the storage stability of such a liquid formulation. We predicted that negatively charged mRNAs inside the LNPs would bind to positively charged ionizable lipids through electrostatic interaction, when the pH was the same as the value for pKa, but we noted interactions with neutral ionizable lipids through hydrogen bonding when pH values rose above that of the pKa [38,39,40]. On the other hand, a recent paper used newly developed coarse-grained Monte Carlo simulations and ^13^C NMR measurement to demonstrate that ionizable lipids neutralize negative charges of encapsulated mRNAs even at higher pH [41]. In any of these provisional theories, the mRNAs and hydrophilic head group of the ionizable lipids are in proximity irrespective of pH conditions. In this respect, we synthesized TOT-28 with a hydrolysis-susceptible ethyl ester moiety in the hydrophilic head region as an indicator of pH and hydration levels in the microenvironment surrounding the mRNA inside LNPs. This enabled us to estimate the microenvironmental alterations that would occur under various storage conditions of pH and temperature and to predict the effects on storage stability.

Our results indicated that different temperatures resulted in different degrees and trends of hydrolysis for TOT-28 following storage at different levels of pH. In general, ester hydrolysis is minimized at neutral pH because it is catalyzed either by protons or hydroxide ions. Therefore, the lower hydrolysis rate at higher pH observed at 25 °C would be reasonable. Since the apparent pKa of TOT-28-LNPs is approximately 6.0, most of the TOT-28 is positively charged at pH 5.0, which leads to an increase in the degree of membrane hydration. The increase in membrane hydration would also contribute to an acceleration of the hydrolysis of the ester bonds in the hydrophilic region of TOT-28. Positively charged ionizable lipids are thought to increase the average intermolecular distance due to the charge repulsion between them, which is consistent with the fact that microviscosity of the TOT-28-LNPs decreased under a pH of 5.0 at 25 °C. On the other hand, the higher hydrolysis of TOT-28 at pH 7.0 that was observed at 4 °C clearly defied expectations. However, the increase in membrane hydration of the TOT-28-LNPs at pH 5.0 (particularly at 4 °C) was clearly limited compared with other LNPs. In addition, the microviscosity of the TOT-28-LNPs increased at 4 °C under acidic conditions, which was opposite to the trend observed at 25 °C. These physicochemical properties suggest that the TOT-28-LNPs are not strongly charged at 4 °C and pH 5.0, which suggests a different internal structure from that at 25 °C. The pH inside the LNPs was estimated to be basic compared with that of the bulk solution because ionizable lipids, which are weak basic substances, exist in high concentrations within the LNPs [17]. This could explain the rapid ester hydrolysis at 4 °C in a pH 7.0 storage buffer. While the details of the structure still require clarification, it is important to note that a neutral pH does not necessarily minimize the hydrolysis of the LNP lipids during long-term storage as a liquid formulation when optimizing storage conditions. In addition, we must emphasize that measurements of the physical properties of LNP membranes under each storage condition are important in order to establish the appropriate storage conditions.

Single-strand RNAs (ssRNAs), which includes mRNAs, are known to be inherently susceptible to degradation via in-line hydrolytic cleavage [42,43]. The mRNAs in the TOT-28-LNPs were scarcely degraded at 4 °C after 8 weeks of storage under all values of pH, whereas TOT-28 inside the mRNAs were degraded with an increase in pH following 2 weeks or more at 25 °C. Jarvinen et al. have reported that the hydrolysis of RNA was least likely to occur at around pH 5.0 and would only gradually proceed as the pH increased [42]. Their results are consistent with our experimental results at 25 °C. As mentioned above, it is hypothesized that either the unprotonated tertiary amines in ionizable lipids promote in-line hydrolysis by removing protons from the ribose 2′ hydroxyl groups of mRNAs or that the highly concentrated unprotonated ionizable lipids near the mRNAs raise the level of pH in the surrounding microenvironment compared with levels in the bulk solution. Any of these conditions would promote the alkaline hydrolysis of the mRNAs by hydroxide ions. We recognize the need to adjust the pH to physiological levels prior to administration, but it could be preferable to store the product in a buffer solution with a pH lower than the apparent pKa of the LNPs in order to maintain stability. Such efforts will be particularly critical for long ssRNAs such as the self-amplifying RNA, which are highly susceptible to inactivation by hydrolysis.

Larson et al. previously reported the effects of pH (from 4 to 8) and temperature (from 4 to 25 °C) on the phase behavior and storage stability of mRNA-loaded MC3-LNPs [44]. Transfection efficiency of the fresh MC3-LNPs in HeLa cells was highest at lower pH and decreased as pH increased. At lower pH, the mRNA-loaded LNPs were clearly more stable compared with the effect at higher pH. Based on the results of Fourier transform infrared spectroscopy (FTIR) and differential scanning calorimetry (DSC), these researchers hypothesized that the mRNA-loaded LNPs had inverse hexagonal structures that retain the transfection ability at lower pH and are more stable than lamellar versions. By contrast, our study indicated that the gene expression level of the fresh TOT-28-LNPs in muscle was the lowest at pH 5.0. Considering that positively charged LNPs are more readily taken up by cells in vitro, our in vivo results may trend differently from that of Larson’s observations. Moreover, differences in the ionizable lipid structure (MC3 vs. TOT-28) would cause the above discrepancy due to the fact that the pH-responsibility of membrane properties between both LNPs was clearly distinguished. In the Larson report, mRNA-loaded LNPs stored at 25 °C for 2 weeks resulted in a complete loss of transfection efficiency [44]. At 4 °C, the MC3-LNPs stored at pH6.0 and over lost transfection efficiency at the 4-week timepoint. On the contrary, our study found that approximately more than 50% of gene expression levels against fresh TOT-28-LNPs were maintained for up to 8 weeks at 4 °C. In addition, the mRNA-loaded LNPs stored at 25 °C showed detectable gene expression for up to 4 weeks. These findings are thought to be due to the structural advantage of TOT-28 which suppresses mRNA inactivation through adduct formation. Considering less than 30% of fragment mRNA, the dropping gene expression level at 25 °C after 4 weeks would be caused by significant hydrolysis of TOT-28.

We have demonstrated that the hydrophilic environment inside mRNA-LNPs responds differently to pH changes in bulk solution depending on the storage temperature, which affects the rates of lipid hydrolysis and gene expression. In general, accelerated testing under elevated temperatures is used to evaluate the consistent long-term storage stability of solutions during storage (i.e., consistent pH) under low temperatures over a short period of time. However, as observed with TOT-28-LNPs, there could be interactions between storage conditions such as temperature and pH. Therefore, the results of accelerated testing might not directly extrapolate to levels of storage stability at 4 °C.

## 5. Conclusions

The hydration degree and microviscosity of the lipid assemblies in LNPs with different ionizable lipid structures exhibited distinct responses to pH changes at specific storage temperatures. In particular, TOT-28-LNPs utilized in the current study showed opposite signs of microviscosity shift in response to pH changes at temperatures between 4 and 25 °C, which suggests that the internal structure at each level of pH differs depending on the temperature. Changes in assembly properties were accompanied by alterations in the hydrolysis rates of TOT-28 and mRNA. The findings in this study suggest the risk of limited temperature extrapolation in accelerated testing. This highlights the usefulness of membrane property evaluations in optimizing storage conditions, and provides ideas for future formulation design.

## Data Availability

The data that support the findings of this study are available from the corresponding author upon reasonable request.

## Supplementary Materials

The supporting information can be downloaded at the following address: https://www.mdpi.com/article/10.3390/pharmaceutics17091194/s1, File S1: Synthesis of TOT-28.
