# Supplementary material for: Examining the Impact of Storage Conditions on the Stability of a Liquid Formulation of mRNA-Loaded Lipid Nanoparticles
Source: Pharmaceutics. 2025 Sep 14;17(9):1194. doi: 10.3390/pharmaceutics17091194 (PMC12473483; doi:10.3390/pharmaceutics17091194)
Supplement: Supplementary file 1 [file pharmaceutics-17-01194-s001.zip › pharmaceutics-3795116-supplementary.pdf]

pharmaceutics

Supplementary Materials: Examining the impact of storage conditions on the stability of a liquid formulation of mRNA-loaded lipid nanoparticles

Mina Sato, Eleni Samaridou, Moritz Beck-Broichsitter, Masatoshi Maeki, Shunsuke Kita, Manabu Tokeshi, Katsumi Maenaka, Hideyoshi Harashima and Yusuke Sato

Synthesis of TOT-28

General Information

All reactions were monitored by thin-layer chromatography (TLC) on pre-coated TLC plates (Millipore, Milwaukee, WI, USA) visualized by UV light (254 nm), phosphomolybdic acid stain, and *p*-anisaldehyde stain. The products were purified using the Biotage Selekt system equipped with an ELSD detector (Biotage Selekt ELSD, Biotage, Tokyo, Japan). The separated fractions were analyzed using the Expression CMS TLC-MS system (Advion Interchim Scientific, Ithaca, NY, USA).

All simple chemicals were purchased from commercial sources and were used without further purification.

^{1}H spectra were obtained using either JEOL ECZ500R or ECZ400 instruments with tetramethylsilane as the internal standard (0 ppm).

The purity of the final products was analyzed using an LCMS-2050 (Shimazu Corporation) with an ELSD-LT III detector (Shimazu Corporation). Separation was carried out using a Shim-pack Arata C18 Column (pore size: 120 Å, particle size: 5 µm, inner diameter: 2.0 mm, length: 50 mm) and a gradient of 70-98% isopropanol/acetonitrile (2:1) in water with 5 mM of ammonium acetate over 8 min and held at 98% isopropanol/acetonitrile (2:1) with 5 mM of ammonium acetate for 3 min at 0.2 mL/min. The final products were diluted in ethanol to ~1,000 ppm and used as the measurement sample. The injection volume was 1 µL and the column temperature was 60 °C.

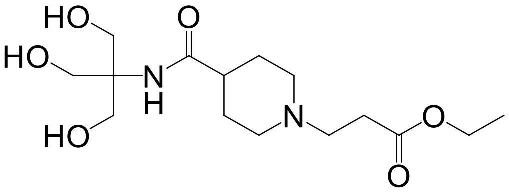

Scheme S1. Synthesis of ethyl 3-(4-((1,3-dihydroxy-2-(hydroxymethyl)propan-2-yl)carbamoyl)piperidin-1-yl)propanoate (TxT-28 head).

4-Piperidinecarboxylic acid (645 mg, 5.0 mmol), ethyl acrylate (1001 mg, 10.0 mmol), and TEA (767 µL, 5.50 mmol) were dissolved in ethanol (2 mL) and were stirred overnight at ambient temperature under an argon atmosphere. The solvent was removed *in vacuo*. The crude product was then suspended in hexane and the suspension was filtered and washed with hexane. The solid residue was dried *in vacuo* and was used for the next step without further purification.

Under an argon atmosphere, 1-(3-ethoxy-3-oxopropyl)piperidine-4-carboxylic acid (988.0 mg, 4.31 mmol) and TEA (959.3 mg, 9.48 mmol) in THF (12 mL) at 0 °C were added dropwise to ethyl chloroformate (514.4 mg, 4.74 mmol) and the mixture was stirred for 30 min at 0 °C. The reaction mixture was added to the tris(hydroxymethyl)-aminomethane (522.1 mg, 4.31 mmol) and DMF (12 mL) mixture and then stirred overnight at ambient temperature. The reaction was monitored via TLC following the addition of TEA (2.64 mL, 18.96 mmol). The solid was filtered off and DMF and THF were evaporated *in vacuo*. The residue of the reaction mixture was loaded onto a normal-phase column (Sfär Amino

D, Biotage) and purified by flash chromatography with a gradient mobile phase of DCM and MeOH. This gave 595.0 mg (41.5%) of **TxT-28 head** as a yellow solid.

^{1}H NMR (500 MHz, CD_3OD, ppm) δ: 1.23 (t, 3H), 1.75 (m, 4H), 2.04 (t, 2H), 2.50 (t, 2H), 2.65 (t, 2H), 2.97 (br, 1H), 3.69 (s, 6H), 4.11 (m, 2H).

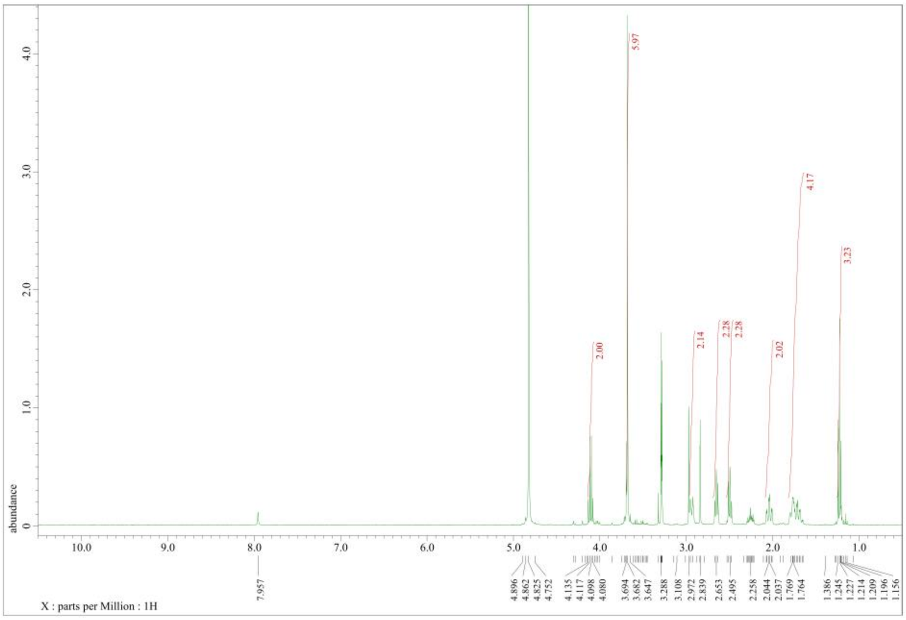

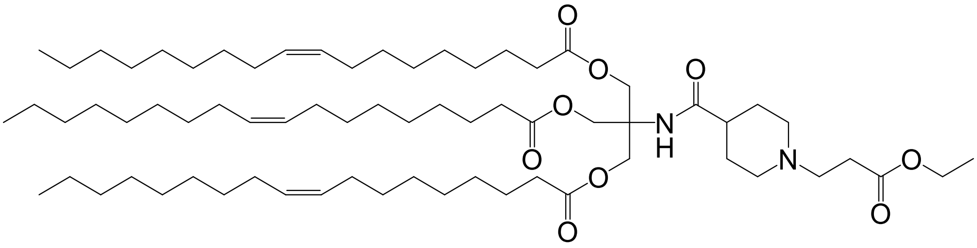

Scheme S2. ethoxy-3-oxopropyl)piperidine-4-carboxamido)-2-((oleoyloxy)methyl)propane-1,3-diyl dioleate (TOT-28).

Oleic acid (1398 mg, 4.95 mmol) was dissolved in anhydrous DCM (4 mL), and **TxT-28 head** (498.6 mg, 1.5 mmol), DMAP (18.3 mg, 0.15 mmol), and EDCI-HCl (1006.4 mg, 5.25 mmol) were added to the mixture. The reaction mixture was stirred at 40 °C under an argon atmosphere overnight. After being concentrated *in vacuo*, the obtained mixture was diluted with water and extracted with EtOAc. The aqueous layer was made alkaline using 8N of NaOHaq. The organic layer was washed with brine, and dried over anhydrous Na_2SO_4. The solution was filtered, and the filtrate was evaporated. The residue was loaded onto a normal-phase column (Sfär Amino D, Biotage) and purified by flash chromatography with a gradient mobile phase of hexane and EtOAc. Additionally, the crude product was loaded onto a reverse-phase column (Sfär Amino C18, Biotage) and purified by flash chromatography with a gradient mobile phase of water with 10 mM ammonium acetate and ACN/iPrOH 1:1 with 10 mM ammonium acetate. This gave 869.8 mg (51.5%) of **TOT-28** as a colorless oil.

^{1}H NMR (500 MHz, $CDCl_3$, ppm) δ: 0.88 (t, 9H), 1.26 (m, 63H), 1.60 (m, 8H), 1.80 (m, 2H), 2.00 (m, 12H), 2.30 (m, 7H), 2.48 (t, 2H), 2.66 (t, 2H), 2.91 (br, 2H), 4.13 (m, 2H), 4.37 (s, 6H), 5.33 (t, 6H), 5.99 (s, 1H).

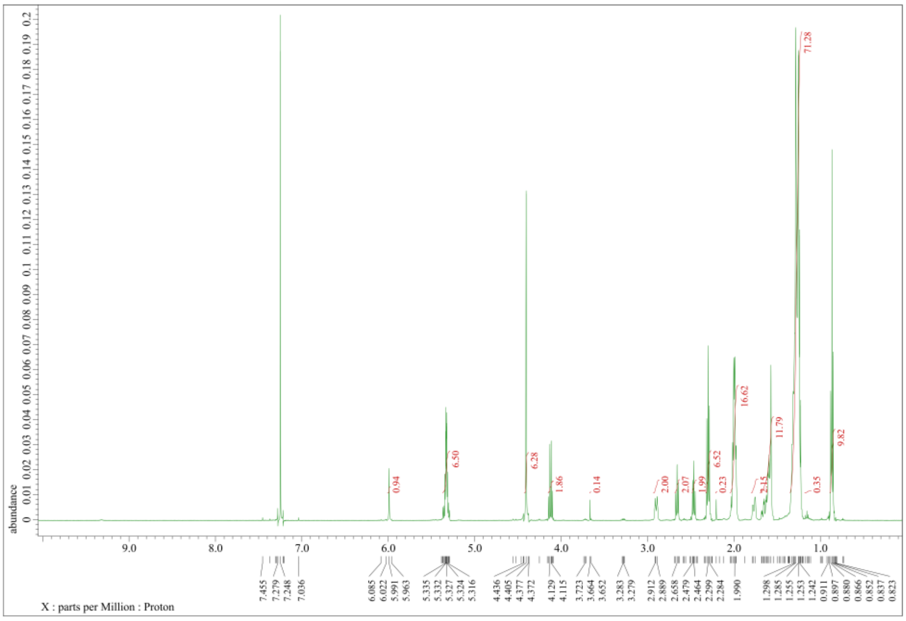

Scheme S3. $H_{124}N_2O_9(M+H)^+$, 1125.93; found, 1125.7 and for $C_{69}H_{124}N_2NaO_9$ $(M+Na)^+$, 1147.93; found, 1047.5.

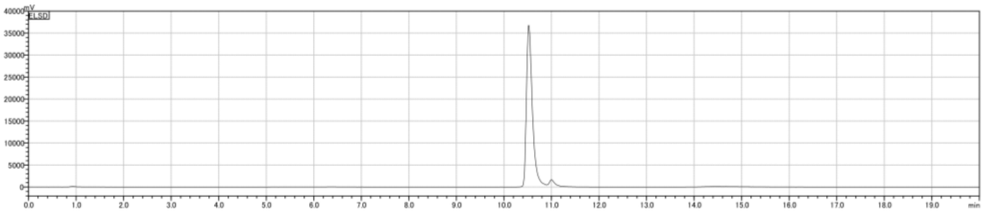

Scheme S4. HPLC/ELSD: TOT-28-peak was detected at 10.5 min and the peak area accounted for 96.9% of the total area.
